# Effects of Chicken Serum Metabolite Treatment on the Blood Glucose Control and Inflammatory Response in Streptozotocin-Induced Type 2 Diabetes Mellitus Rats

**DOI:** 10.3390/ijms24010523

**Published:** 2022-12-28

**Authors:** Xuan Hu, Xueming Liu, Yujiao Guo, Yi Li, Zhengfeng Cao, Yu Zhang, Yang Zhang, Guohong Chen, Qi Xu

**Affiliations:** Jiangsu Key Laboratory for Animal Genetic, Breeding and Molecular Design, Yangzhou University, Yangzhou 225009, China

**Keywords:** chicken, streptozotocin-induced rats, DL-arginine, blood glucose, inflammatory response

## Abstract

Chickens can live healthy without adverse effects despite high blood glucose levels. However, the blood biomolecules responsible for maintaining chronic hyperglycemia are unknown. Here, the effects of chicken serum metabolite treatment on blood glucose control and inflammatory response in streptozotocin (STZ)-induced Type 2 Diabetes Mellitus (T2DM) rats were investigated. First, chicken serum treatment reduced the advanced glycation end-products (AGEs) and blood glucose levels in STZ-induced T2DM rats. Second, insulin/glucose-induced acute hypoglycemic/hyperglycemic chickens and the blood biomolecules were screened via nontargeted ultra-performance liquid chromatography with mass spectroscopy (UPLC-MS), identifying 366 key metabolites, including DL-arginine and taurine, as potential markers for chronic hyperglycemia in chickens. Finally, DL-arginine functions for blood glucose control and inflammatory response were evaluated. We found that DL-arginine reduced the levels of blood glucose and AGEs in STZ-induced T2DM rats. In addition, DL-arginine treatment upregulated the glucose transporter type 4 (*GLUT4*) expression in the muscles and downregulated the advanced glycation end products receptor-1 (*AGER1*) expression in the liver and nuclear factor-κB (*NF-κB*) and mitogen-activated protein kinase (*MAPK*) expression in the pancreas and thymus tissues. Overall, these results demonstrate that serum metabolite of DL-arginine could maintain blood glucose homeostasis and suppress the inflammatory response in chickens. Therefore, DL-arginine may be a novel target for developing therapeutic agents to regulate hyperglycemia.

## 1. Introduction

The normal plasma glucose concentration in many birds is 1.5 to 2 times higher than in mammals of similar body weight [1]. Blood glucose levels are regulated by hormones such as insulin and glucagon. Birds have significantly lower plasma insulin levels, but higher glucagon levels than mammals and are resistant to the hypoglycemic effects of insulin [2,3]. In addition, blood glucose levels in the body are not only related to food carbohydrate intake, liver glycogen decomposition, and non-sugar substance gluconeogenesis but may also be related to its oxidative breakdown and glycogen synthesis. Both hummingbirds and shrew-shaped bats prefer high-sugar diets, so they tend to have higher blood glucose levels [4]. Studies have shown that hummingbirds and bats can use hyperglycemia to provide fuel for flight [5,6]. However, chickens have lost the ability to fly and migrate, hence their need to maintain high glucose concentration, together with the physiological function involved remains unclear.

In mammals, chronic hyperglycemia is almost always associated with severe adverse effects due to the increased production of harmful by-products, such as reactive oxygen species and advanced glycation end products (AGEs); however, birds do not show any related abnormalities [7]. Additionally, unlike in mammals, AGEs receptors (RAGE) do not exist in poultry genomes [8], which is possibly attributed to the high blood glucose levels not causing the same complications in chickens as observed in patients with hyperglycemia [9], a condition that promotes the production of AGEs through the non-enzymatic reduction of sugars (glucose) and proteins. AGEs are harmful compounds that form when proteins bind to sugars in the blood via the Maillard or carbonyl ammonia reaction, which is the reaction between carbonyl compounds (reducing sugars) and amino compounds (amino acids and proteins) [7]. Thus, lowering the AGE levels in the body can effectively prevent the adverse effects caused by hyperglycemia.

The control or reversal of diabetic complications through supplementation with carbonyl scavengers has been repeatedly attempted in human and animal models, mainly using taurine [10,11], arginine, and other amino acids [12,13], as well as carnosine [14,15], ergosulfonic acid [16], thiamine and its analogs [17,18], pyridoxamine [19] and aminoguanidine [20]. Chicken blood contains various proteins, amino acids, and trace elements. The total concentration of free amino acids in bird plasma is approximately 7–8 mmol/L, whereas that in human plasma is only 3–4 mmol/L [21]. For instance, the total average concentration of asparagine, glutamine, lysine, serine, threonine, taurine, and arginine in humans is approximately 1000 μmol/L, whereas their total average concentration in birds is approximately 4000 μmol/L [21]. Therefore, we speculate that carbonyl scavengers in the chicken serum inhibit the Maillard reaction, reduce AGEs production in vivo, and regulate blood glucose homeostasis.

Recently, L-arginine, as a carbonyl scavenger, has been implicated in the regulation of blood glucose and insulin sensitivity. As noted by Monti LD. et al., the administration of L-arginine could delay the development of type 2 diabetes mellitus (T2DM) for a long period [22]. A more important fact is that L-arginine may have the potential to prevent and/or relieve type 2 diabetes by restoring insulin sensitivity [23,24]. Metabolic syndrome, glucose level dysregulation, and diabetes are one of the greatest health concerns worldwide. Chickens have the physiological characteristics of hyperglycemia without adverse symptoms, but the blood biomolecules responsible for maintaining chronic hyperglycemia are unknown.

In the present study, we investigated the effects of chicken serum treatment on blood glucose control and inflammatory responses in streptozotocin (STZ)-induced Type 2 Diabetes Mellitus (T2DM) rats. DL-arginine was identified as a key metabolite for blood glucose regulation using nontargeted ultra-performance liquid chromatography with mass spectroscopy (UPLC-MS), and its functions in glucose control and inflammatory responses were also determined in STZ-induced T2DM rats. Our findings not only revealed the physiological mechanism underlying chronic hyperglycemia in chickens but also elucidated the role of DL-arginine in regulating hyperglycemia tolerance, which hinted that DL-arginine might be a novel target for developing therapeutic agents to regulate hyperglycemia.

## 2. Results

### 2.1. STZ-Induced T2DM Rats and Insulin/Glucose-Induced Acute Hypoglycemic/Hyperglycemic Chickens

**Figure 1:**
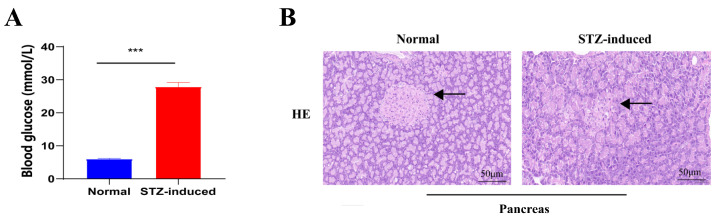
Streptozotocin (STZ)-induced type 2 diabetes mellitus (T2DM) rat model. Eight-week-old male Sprague-Dawley (SD) rats were fed high-fat diets for 4 weeks and administrated STZ (35 mg/kg BW) via intraperitoneal injection, 1 week after STZ infusion, and rats were assessed using the following tests. (**A**) Blood glucose levels were determined in venous blood samples obtained from alert fasted animals using a glucometer. (**B**) Pancreas histology was studied using serial 4 µm hematoxylin/eosin-stained (HE) sections, which were observed via light microscopy, focusing on islet structures (Scale bar = 50 μm). All values are presented as the mean ± SE of *n* = 6 rats per group, with *** *p <* 0.001, as determined by Student’s *t*-tests.

**Figure 2:**
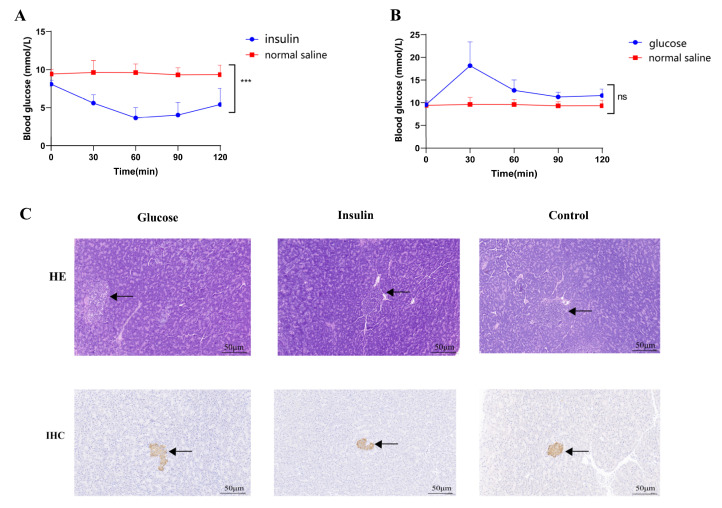
Acute hypoglycemia and hyperglycemia, induced by insulin/glucose, in chickens. (**A**) Acute hypoglycemic chickens using subcutaneous insulin injection (mmol/L). (**B**) Acute hyperglycemic chickens using oral glucose administration (mmol/L). (**C**) Morphology of pancreatic islets in different treatment groups stained with hematoxylin and eosin (HE) and β-cells were characterized by immunohistochemistry (IHC). Sections (5 μm) were cut from 4% paraformaldehyde-fixed tissue and treated with purified anti-mouse IRS1 antibody (Scale bar = 50 μm). The HE staining arrow refers to the morphology of the pancreatic islets, and the IHC arrow refers to the positive signal of the islet β cells (yellow). All values of (**A**,**B**) are mean ± SE *n* = 6 chickens per group, with *** *p <* 0.001, determined by one-way ANOVA. “ns” is “no significant”.

To test the therapeutic effects of chicken serum on STZ-induced T2DM rats, we established a hyperglycemia rat model treated with STZ combined with a high-fat diet (HFD). The blood glucose levels in STZ-induced T2DM rats get became close to 30 mmol/L, which was significantly higher than that in normal rats (Figure 1A), and pancreas histology revealed smaller islets (Figure 1B). In addition, acute hypoglycemic and hyperglycemic chickens were induced via the subcutaneous injection of insulin and glucose oral administration, respectively. Acute hypoglycemic chickens showed a marked drop in blood glucose levels, decreasing to 3.84 mmol/L after 60 min of treatment (Figure 2A). By contrast, the blood glucose levels sharply increased in 30 min glucose treatment at 19.54 mmol/L and returned to normal after 120 min in acute hyperglycemic chickens (Figure 2B). Meanwhile, no significant fluctuations in blood glucose were observed in the control birds. Furthermore, the pancreatic tissue injury was examined in the acute hypoglycemic and hyperglycemic chickens via histological sectioning and immunohistochemistry. There was no obvious tissue and cell injury in the pancreatic islets compared with the control chickens (Figure 2C).

### 2.2. Effects of Chicken Serum Treatment on Blood Glucose Control and Inflammatory Response

**Figure 3:**
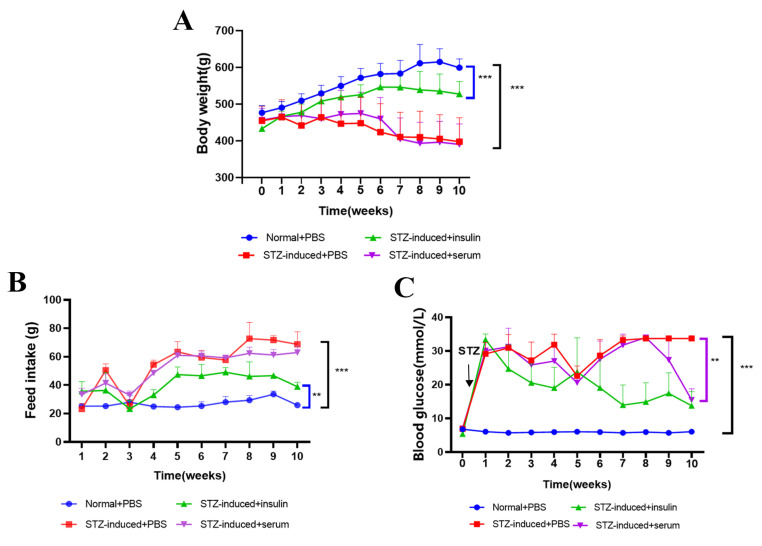
Effects of chicken serum treatment on feed intake, body weight, and blood glucose of rats. (**A**) The body weight of each group was determined every week. (**B**) The feed intake of each group was determined every week (**C**). Blood glucose levels of each group were after fasting for 3 h determined every week. All values of (**A**–**C**) are mean ± SE *n* = 6 rats per group, with ** *p <* 0.01, *** *p <* 0.001, determined by one-way ANOVA.

To investigate the therapeutic effect of chicken serum on STZ-induced T2DM rats, feed intake, body weight, and blood glucose levels were evaluated. We found that the body weights of chicken serum-treated STZ-induced T2DM rats continued to decrease and were significantly lower than those in normal rats or with insulin treatment after 10 weeks (Figure 3A). In contrast, higher feed intake was observed in STZ-induced T2DM rats treated with chicken serum compared with the normal rats. We also noticed that the serum-treated rats showed consistently lower feed intake after eight weeks (Figure 3B). Suggesting that the rats maintained high metabolic rates while maintaining hyperglycemic substances in vivo. In addition, blood glucose levels were similar in the early stages between serum-treated or untreated STZ-induced T2DM rats. However, blood glucose levels decreased sharply after 8 weeks of treatment and then returned to normal levels 10 weeks later (Figure 3C).

Moreover, serum fructosamine and AGE levels were measured. There was no significant difference in the fructosamine contents between the chicken serum-treated and untreated STZ-induced T2DM rats. Similarly, no significant differences in alanine aminotransferase, aspartate aminotransferase, aspartate/alanine, and alkaline phosphatase levels were observed (Supplementary Figure S2). However, the AGEs content was significantly reduced in the chicken serum-treated rats, suggesting that chicken serum can inhibit AGEs production (Supplementary Figure S1).

### 2.3. Key Metabolites Screening for Glucose Regulation in the Chicken Serum

**Figure 4:**
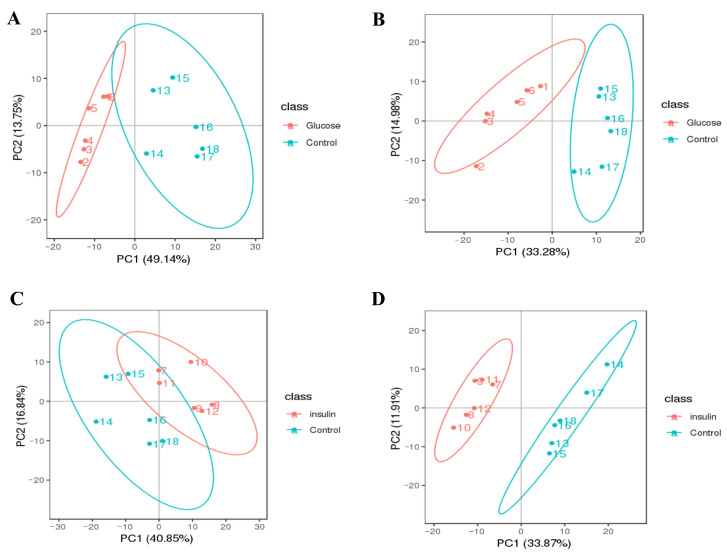
Principal component analysis of QC samples of serum metabolites from acute hyperglycemic/hypoglycemic chickens and control birds. (**A**) Acute hyperglycemic chickens and control birds PCA plot positive-ion pattern. (**B**) Acute hyperglycemic chickens and control birds PCA plot negative ion pattern. (**C**) Acute hypoglycemic chickens and control birds PCA plot positive ion pattern. (**D**) Acute hypoglycemic chickens and control birds PCA plot negative ion pattern. Abscissa PC1 and ordinate PC2 in the figure indicate the scores of principal components ranked first and second, respectively, and scatter in different colors indicates the samples of different experimental groups, with ellipses of 95% confidence intervals.

After LC-MS/MS detection, a total of 28,711 positive and 7216 negative ion modes were obtained. With the coefficient of variation set to <30% as the filter [25], 1376 effective metabolites were identified, including 429 positive and 259 negative ion modes per group (Supplementary Figure S3). The QC values were close to 1, indicating that the LC-MS/MS system has very good stability, and the quality of the collected data was high (Supplementary Figure S4). Multivariate analysis software was used to evaluate the data. Principal component analysis was performed to observe the overall distribution trends among the three treatment groups. Generally, the two coordinates were >50% distributed, and the model interpretation rate was good, indicating that the principal component analysis model was ideal. In addition, the samples were within the 95% confidence interval, and the different groups had their own distribution regions (Figure 4). The metabolites of the acute hyperglycemic chickens were easily distinguished from those of the control birds, whereas the metabolites of the acute hypoglycemic chickens and control birds partially overlapped.

### 2.4. Key Metabolites Identification for Glucose Regulation in Hyperglycemic Chickens

**Figure 5:**
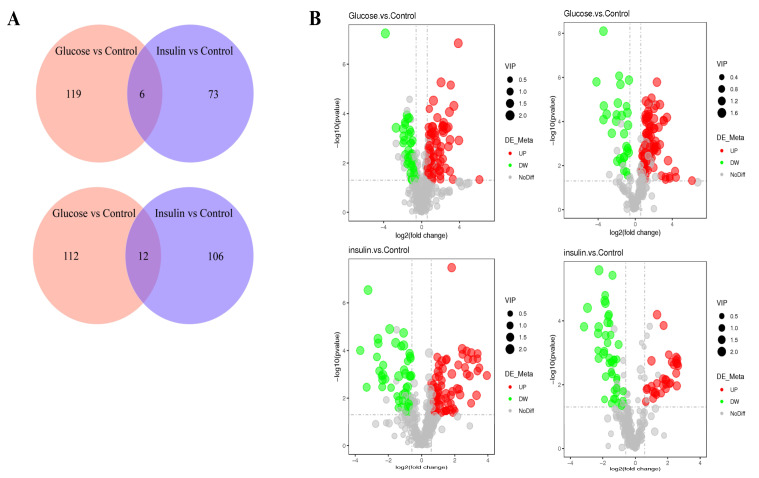
Differential metabolite Venn plots and Volcano plots of acute hyperglycemic/hypoglycemic chickens and control birds. (**A**) Differential metabolite Venn plots for acute hyperglycemic/hypoglycemic chickens and control model. (**B**) Differential metabolites Volcano plots for acute hyperglycemic/hypoglycemic chickens and control birds. Venn diagrams can visually compare common and unique differential metabolites between different groups and show the relationship between multiple groups of differential metabolites. The abscissa represents the fold change of difference (log2 Fold Change) of metabolites in different groups, the ordinate represents the significance level of difference (−log10 *p*-value), each point in the volcano plot represents a metabolite, significantly up-regulated metabolites are represented by red points, significantly down-regulated metabolites are represented by green points. The size of the dot represents VIP value.

**Figure 6:**
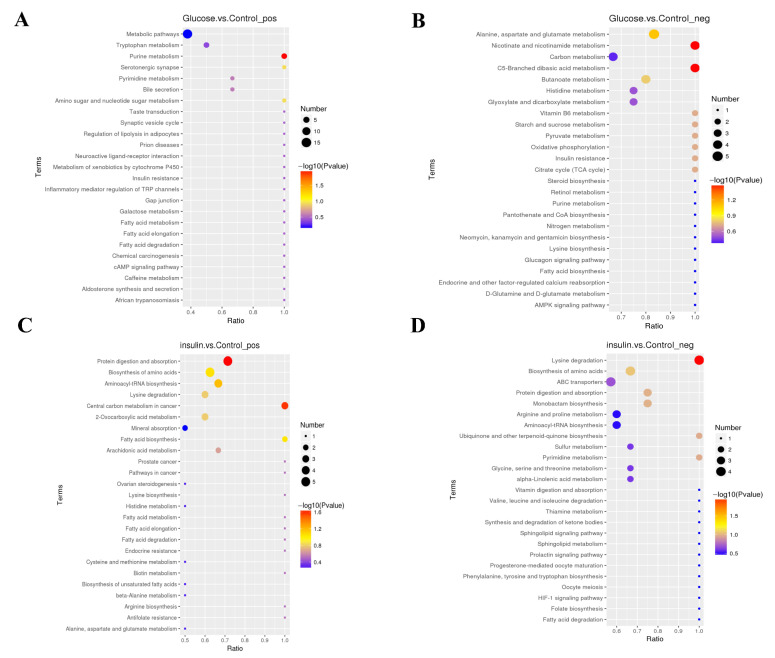
KEGG Enrich scatterplot of acute hyperglycemic/hypoglycemic chickens and control birds. (**A**) Acute hyperglycemic chickens and control birds KEGG Enrich scatterplot positive ion pattern. (**B**) Acute hyperglycemic chickens and control birds KEGG Enrich scatterplot negative ion pattern. (**C**) Acute hypoglycemic chickens and control birds KEGG Enrich scatterplot positive ion pattern. (**D**) Acute hypoglycemic chickens and control birds KEGG Enrich scatterplot negative ion pattern. The abscissa in the figure is x/y (number of differential metabolites in the corresponding metabolic pathway/total number of metabolites identified in the pathway), and a larger value indicates a higher enrichment of differential metabolites in the pathway. The color of the dots represents the *p*-value value of the hypergeometric test, and the smaller the value, the greater and statistically significant the reliability of the test.

**Figure 7:**
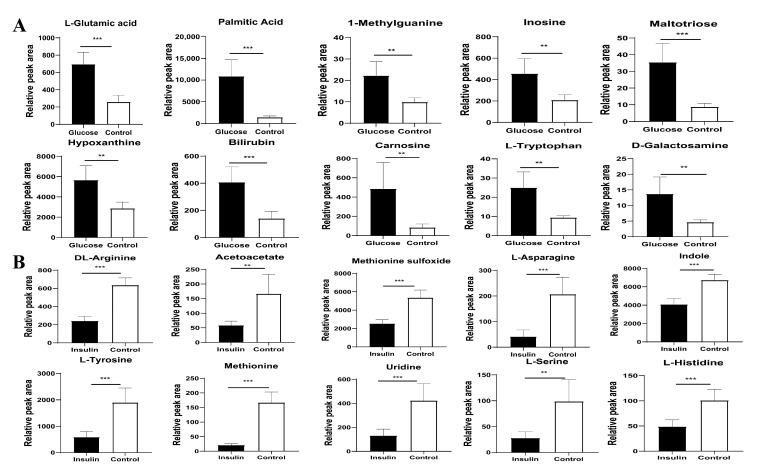
Differential metabolites of acute hyperglycemic/hypoglycemic chickens and control birds. (**A**) Peak area of representative key metabolites regulating blood glucose rise. (**B**) Peak area of representative key metabolites regulating blood glucose decline. All values of (**A**,**B**) are mean ± SE *n* = 6 chickens per group, with ** *p <* 0.01, *** *p <* 0.001, determined by Student’s *t*-test.

Based on the set threshold (Variable Importance in the Projection [VIP] > 1.0, 0.667 < Fold Change [FC] > 1.5, and *p*-value < 0.05) [26,27], 446 differentially expressed metabolites were identified from the 1376 effective metabolites (Figure 5, Table 1). Correlation analysis with phenotypic parameters, such as blood glucose and serum biochemical indexes, revealed that 366 metabolites were significantly correlated with blood glucose regulation (*p* < 0.05) (Supplementary Figure S5). To clearly understand the changes in the steady-state differentially expressed metabolites, the peak area was analyzed using hierarchical cluster analysis, revealing that DL-arginine metabolites are involved in the homeostatic regulation of glucose metabolism in hyperglycemic chicken (Supplementary Figure S6). Subsequent Kyoto Encyclopedia of Genes and Genomes (KEGG) pathway enrichment analysis of the differentially expressed biomarkers showed that the metabolites were mainly involved in protein digestion and absorption, insulin resistance (IR), glycolysis, citric acid cycle, carbon metabolism, pyruvate metabolism, glucagon signaling pathway, arginine and proline metabolism, and other metabolic pathways (Figure 6). Quantitative analysis of serum metabolites in the acute hyperglycemic and hypoglycemic chickens using nontargeted metabolomics revealed that L-glutamic acid, palmitic acid, 1-methylguanine, inosine, maltose, hypoxanthine, bilirubin, inosine, L-tryptophan, and D-aminogalactose, which regulate the increase of blood glucose levels in chickens, were significantly different from those in the control group (*p* < 0.01). Likewise, the amino acids regulating blood glucose decrease in chickens, specifically DL-arginine, acetyl acetate, methionine sulfoxide, L-asparaginamide, indole, L-tyrosine, methionine, uridine, L-serine, and L-histidine, were significantly different from those in the control group (*p* < 0.01), (Figure 7).

### 2.5. Effects of DL-Arginine on Blood Glucose Control and Insulin Sensitivity in STZ-Induced T2DM Rats

**Figure 8:**
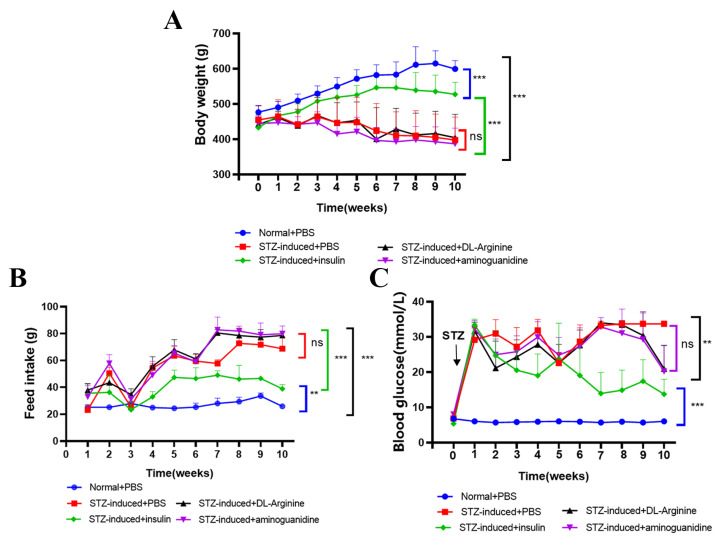
Effects of DL-arginine treatment on feed intake, body weight, and blood glucose of rats. (**A**) The body weight of each group was determined every week. (**B**) The feed intake of each group was determined every week. (**C**) Blood glucose levels of each group were after fasting for 3 h determined every week. All values of (**A**–**C**) are mean ± SE *n* = 6 rats per group, with ** *p <* 0.01, *** *p <* 0.001, determined by one-way ANOVA.

**Figure 9:**
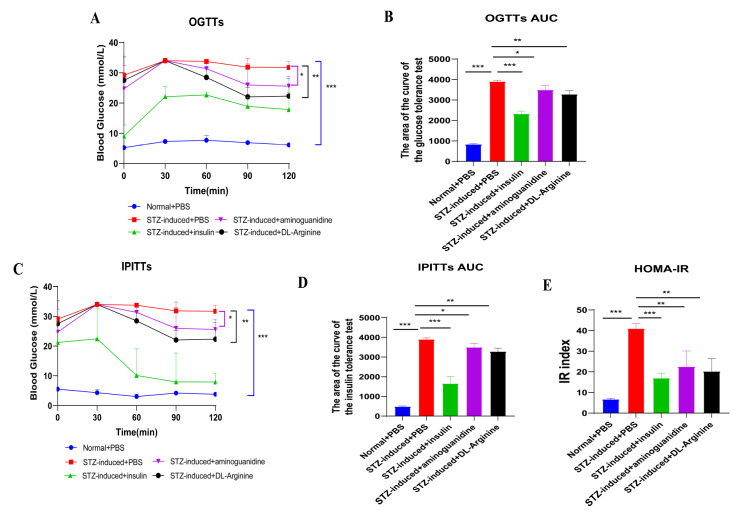
Effect of DL-arginine on rats’ glucose tolerance and insulin tolerance. (**A**) Rat glucose tolerance tests (OGTTs). Individual glucose tolerance was assessed by oral glucose tolerant tests. The fasted rats were oral administration of 2 g of glucose/kg body weight (BW). (**B**) The area under the curve for rat OGTTs. (**C**) Rat insulin tolerance tests (IPITTs). Individual insulin tolerance was evaluated by subcutaneous injection insulin tolerance tests by oral administration of 2 g glucose/kg BW immediately followed by subcutaneous insulin injection at a dose of 2 IU/kg BW; blood glucose levels were detected at 0, 30, 60, 90, and 120 min and compared to that at 0 min. (**D**) The area under the curve for rat IPITTs. (**E**) IR index of each group, HOMA-IR index = (FBG [in mmol/L] × FINS [in units/L])/22.5. All values are mean ± SE *n* = 6 rats per group, with * *p <* 0.05, ** *p <* 0.01, *** *p <* 0.001, determined by (**B**,**D**,**E**) by one-way ANOVA, determined with (**A**,**C**) by repeated ANOVA measurement.

To investigate the therapeutic effect of DL-arginine on STZ-induced T2DM rats, the feed intake, body weight, and blood glucose levels in STZ-induced T2DM rats treated with DL-arginine were evaluated. STZ-induced T2DM rats treated with DL-arginine and aminoguanidine did not regain their body weight and still behaved as emaciated as STZ-induced T2DM rats (Figure 8A). However, feed intake tended to be normal in DL-arginine-treated rats (Figure 8B). For blood glucose levels, the DL-arginine treatment did not cause any change in the early stage, but the blood glucose content began to decrease after 7 weeks of treatment and was significantly lower than that in STZ-induced T2DM rats at 10 weeks (*p* < 0.05), (Figure 8C). The glucose and insulin tolerance of STZ-induced T2DM rats treated with DL-arginine were also evaluated. The results showed that DL-arginine had a significant favorable effect on glucose tolerance (*p* < 0.01) (Figure 9A,B). In addition, insulin sensitivity was significantly improved in DL-arginine-treated STZ-induced T2DM rats (*p* < 0.01) (Figure 9C–E).

### 2.6. Effects of DL-Arginine on the Expression of Genes Involved in Glucose Metabolism in STZ-Induced T2DM Rats

**Figure 10:**
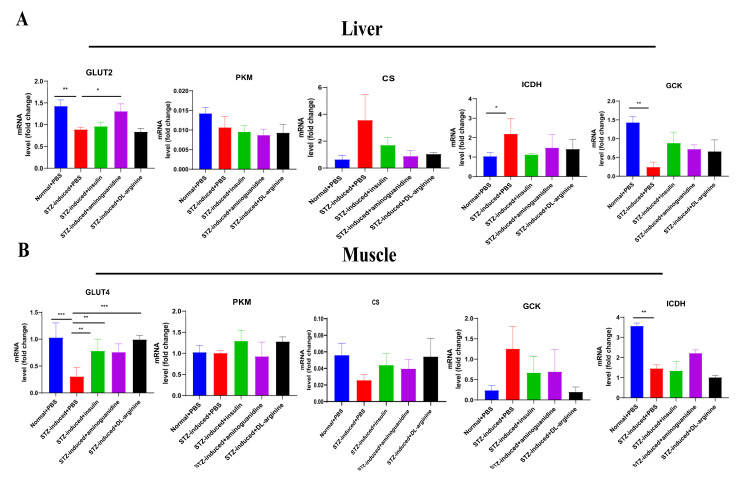
Effects of DL-arginine on the content of glucose metabolism-related enzymes (*GLUT2/4, PKM, CS, GCK, ICDH*) in the liver and muscle of rats. (**A**) Expression of glucose transporters 2 *(GLUT2)*, pyruvate kinase M (*PKM*), glucokinase (*GCK*), citrate synthase (*CS*), and isocitrate dehydrogenase (*ICDH*) mRNA in the liver was analyzed by quantitative RT-PCR. (**B**) Expression of *GLUT4, PK, CS*, and *ICDH* mRNA in muscle was analyzed by quantitative RT-PCR. All values of (**A**,**B**) are means ± SE; *n* = 3 per group, with * *p <* 0.05, ** *p <* 0.01, *** *p <* 0.001, determined by one-way ANOVA.

The effects of DL-arginine on expression levels of genes coding for glucose transporters (GLUTs) and enzymes associated with glucose metabolism were detected. The results showed DL-arginine treatment resulted in significantly upregulated GLUT type 4 (*GLUT4*) expression in the skeletal muscle compared with STZ-induced T2DM rats (*p* < 0.01), while *GLUT2* expression had no significant change in the liver (*p* > 0.05). Furthermore, the expression of glucose metabolism-related genes, specifically glucokinase, pyruvate kinase, citrate synthase, and isocitrate dehydrogenase, were not significantly altered in the liver and skeletal muscle after DL-arginine treatment (*p* > 0.05). These results indicate that DL-arginine can promote glucose intake and cellular metabolism in tissue cells by enhancing *GLUT4* expression in the muscles (Figure 10).

### 2.7. Effects of DL-Arginine on the Pathological Injury and Expression of Genes Involved in Inflammatory Response in STZ-Induced T2DM Rats

**Figure 11:**
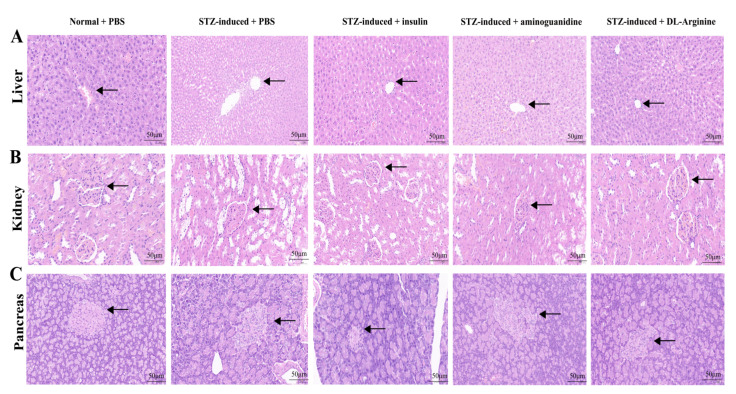
HE sections of the liver, kidney, and pancreas in different treatment groups of rats. (**A**) HE staining was used to detect the degree of injury and recovery in the liver sections. The arrow refers to the distribution of lipid droplet accumulation. (**B**) HE staining was used to detect the degree of injury and recovery in the kidney sections. The arrow refers to the distribution of lipid droplet accumulation glomeruli. (**C**) HE staining was used to detect the degree of injury and recovery in the pancreatic sections. The arrow refers to the distribution of pancreatic islets. (Scale bar = 50 μm).

The effects of DL-arginine on the AGEs and its precursor (fructosamine) were detected in STZ-induced T2DM rats. DL-arginine treatment led to significantly downregulated fructosamine (*p* < 0.05) and AGEs (*p* < 0.01) contents in comparison with STZ-induced T2DM rats (Supplementary Figure S7). Also, the pathological injury of the liver, kidney, and pancreas. In liver tissue, a more complete hepatic cell structure and less lipid droplet accumulation were observed in DL-arginine-treated STZ-induced T2DM rats compared with STZ-induced T2DM rats (Figure 11A). In kidney tissue, the glomeruli were swollen and infiltrated with lymphocytes in DL-arginine or aminoguanidine-treated STZ-induced T2DM rats, but the vacuolization of the renal tubules significantly relieved in DL-arginine-treated STZ-induced T2DM rats, suggesting that DL-arginine could alleviate the renal lesions in STZ-induced T2DM rats to some extent (Figure 11B). In pancreas tissue, we also found pancreas islet morphology gradually recovered in aminoguanidine or DL-arginine treatments (Figure 11C). Overall, the results of the histological examination further confirmed the therapeutic effect of DL-arginine on STZ-induced T2DM rats.

**Figure 12:**
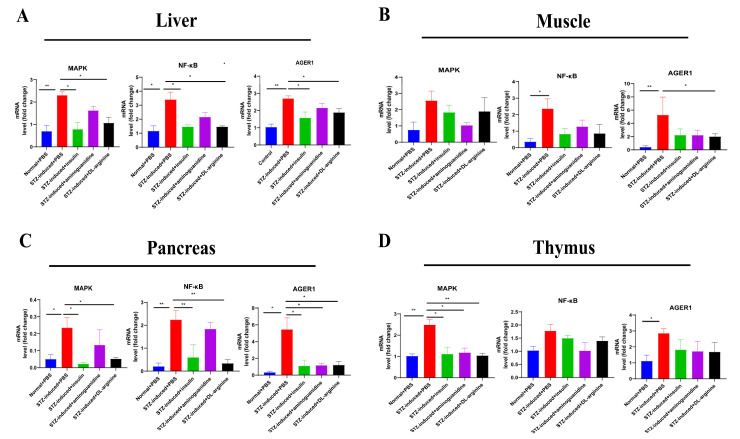
Effects of DL-arginine on the expression of inflammatory genes *(MAPK, NF-κB, AGER1)* in rat tissues. (**A**) Expression of mitogen-activated protein kinase (*MAPK*), nuclear factor- κB (*NF-κB*), and AGE receptor 1 (*AGER1*) mRNA in the liver was analyzed by quantitative RT-PCR. (**B**) Expression of *MAPK, NF-κB,* and *AGER1* mRNA in muscle was analyzed by quantitative RT-PCR. (**C**) Expression of *MAPK, NF-κB, and AGER1* mRNA in the pancreas was analyzed by quantitative RT-PCR. (**D**) Expression of *MAPK, NF-κB*, and *AGER1* mRNA in the thymus was analyzed by quantitative RT-PCR. All values of (**A**–**D**) are means ± SE; *n* = 3 per group, with * *p <* 0.05, ** *p <* 0.01, determined by one-way ANOVA.

Furthermore, the effects of DL-arginine on the inflammatory-related genes expression in the liver, skeletal muscle, pancreas, and thymus of STZ-induced T2DM rats were detected, including mitogen-activated protein kinase (*MAPK*), nuclear factor- κB (*NF-κB*), and AGE receptor 1 (*AGER1*). The expression of *MAPK*, *NF-κB*, and *AGER1* in the liver was significantly lower in the DL-arginine-treated STZ-induced T2DM rats than in the STZ-induced T2DM rats (*p* < 0.05) (Figure 12A). In contrast, DL-arginine treatment did not significantly affect the expression of *MAPK* and *NF-κB* in the skeletal muscle. In addition, the lower expression of *AGER1* in the skeletal muscle (Figure 12B), the lower *MAPK*, *NF*-*κB*, and *AGER1* in the pancreas (Figure 12C), and the lower *MAPK* in the thymus were observed in STZ-induced T2DM rat group treated with DL-arginine (*p* < 0.05), (Figure 12D).

## 3. Discussion

Birds not only have high blood glucose concentrations but are also resistant to insulin-mediated glucose regulation [1,2]. Glycosylation of proteins and subsequent production of AGEs are the most important factors leading to hyperglycemia-induced pathological changes [9]. However, there are no studies investigating the AGEs content and potential side effects of this chronic condition in chickens. Our experiments assessed whether chicken serum treatment mitigated STZ-induced T2DM in rats. The results showed that the changes in body weight and feed intake between the control and treated groups were the same, indicating the maintenance of hyperglycemic substances and hypermetabolism in chickens. Consistently lower feed intake was also observed after eight weeks in serum-treated rats. This fact might be related to the drop in blood glucose levels after eight weeks of age. Notably, the weight loss continued over time [28], which may be related to reduced glucose utilization for energy production and enhanced decomposition of fat and protein. Long-term monitoring of blood glucose results revealed that chicken serum could significantly reduce the blood glucose content in STZ-induced T2DM rats, and based on their mental status, we predicted that the possible side effects were correspondingly reduced, as evidenced by the considerable decrease in AGEs levels. Therefore, we speculated that a particular biomolecule in the chicken serum induces a protective effect against high AGEs production.

To identify the biomolecule in the chicken serum that can reduce AGEs in STZ-induced T2DM rats, we screened the key metabolic markers of blood glucose regulation in the acute hyperglycemic/hypoglycemic chickens and control birds using nontargeted UPLC-MS and multivariate statistical analysis. Metabolomics is a method for collecting detailed information on low molecular weight metabolites within biological systems, thereby describing the health status of a particular organism [29]. A total of 366 metabolites were identified as potential markers involved in the homeostatic regulation of blood glucose in hyperglycemic chickens. Among these, DL-arginine and taurine are known to decrease blood glucose levels. Because of the limited knowledge in this area, the biological function of the remaining biomarkers is still unclear and requires follow-up studies.

The blood’s seven most active free amino acids are aspartic acid, glutamine, lysine, serine, threonine, taurine, and arginine. Taurine and asparagine levels in birds are 6–11 times higher than those in humans, whereas methylglyoxal (MG) -specific arginine levels are almost four times higher than in humans. The concentration difference between birds and humans is quite significant for some amino acids, such as taurine, asparagine, and arginine [21]. Free fatty acids in chickens can be used as carbonyl scavengers and glycosyltransferases, in which the adducts formed between carbonyl groups and their scavengers are eliminated in an active and irreversible process, thereby facilitating the removal of unwanted compounds in the plasma and effectively preventing the formation of AGEs through the Maillard reaction. Therefore, we used DL-arginine, a key metabolic marker for glucose regulation in the chicken serum, to investigate its therapeutic effects on STZ-induced T2DM rats.

We further investigated the effects of DL-arginine on the blood glucose control and inflammatory response of STZ-induced T2DM rats and discovered that DL-arginine treatment negatively affected the body weight and feed intake of STZ-induced T2DM rats. This may be attributed to the cytotoxicity of STZ that leads to rapid β-cell failure or elevated levels of leptin and adiponectin in diabetic rats [30], which mimics the pathophysiology of lean diabetes patients with severe insulin secretion defects compared with individuals with obesity [31]. Alternatively, excess adiposity with reduced muscle mass should be assessed because the ratio of fat percentage to total body weight is more important than total body weight [31,32]. Notably, the blood glucose levels significantly decreased after 10 weeks of treatment, indicating that DL-arginine can ameliorate STZ-induced symptoms and regulate glucose metabolism.

Aminoguanidine also showed a weak hypoglycemic effect on STZ-induced T2DM rats, which may be related to the inhibition of AGEs production. Additionally, the OGTT results indicated that glucose metabolism in STZ-induced T2DM rats was significantly improved by DL-arginine administration, whereas the IPITT results showed a significant improvement in insulin sensitivity after DL-arginine treatment. The HOMA-IR index in the DL-arginine group was considerably lower than in the normal group, and exogenous insulin did not influence individual insulin sensitivity. As an effective biomarker for predicting insulin sensitivity and IR [33], the HOMA-IR index after treatment implies that DL-arginine enhanced insulin sensitivity and alleviated the IR in STZ-induced T2DM rats, resulting in accelerated glucose metabolism. Similarly, Monti et al. have demonstrated the beneficial effects of L-arginine on glucose tolerance and insulin sensitivity, and secretion in mitigating the progression to T2DM [22]. Other findings have also suggested the involvement of L-arginine in multiple NO-dependent pathways that regulate glucose and insulin homeostasis [34,35].

Long-term hyperglycemia can damage various organ systems in patients with diabetes. Over time, the risk of developing severe diabetic complications, such as diabetic nephropathy, increases [36]. Notably, DL-arginine significantly reduced the fructosamine and AGE levels and alleviated the tissue damage in the liver, kidneys, and pancreas of STZ-induced T2DM rats. This data indicates that the DL-arginine treatment did not significantly damage healthy tissues, providing partial evidence for its safety in future clinical applications. L-arginine is a precursor of nitric oxide production by endothelial cells through the action of endothelial nitric oxide synthase. Stimulation of NO production in endothelial cells by insulin improves endothelial function and negatively affects insulin resistance [37]. L-arginine can inhibit hemoglobin glycosylation and lipid peroxidation in vivo, which may prevent complications associated with diabetes [38].

Mechanically, we found DL-arginine might promote glucose uptake and cellular metabolism by enhancing *GLUT4* expression in the muscle. The liver can also uptake glucose in an insulin-dependent manner through the glucose-sensitive transporter *GLUT2* [39], whose expression could be induced by STZ. However, we observed no significant effect on the expression of hepatic glucose metabolism-related enzymes. NF-κB, an important transcription factor regulating immune function, is involved in the pathology of several diseases, including diabetes [40,41]. In hyperglycemia, owing to the inevitable production of reactive oxygen species, chronic oxidative stress activates a series of stress-sensitive signaling pathways, including NF-κB, thereby increasing the expression of several gene products that ultimately cause cell damage and significantly influence the etiology of diabetic complications [41,42]. For example, the MAPK pathway is linked to several pathogenic responses, including inflammation and oxidative stress. Supporting evidence has shown that MAPK signaling pathways are also critically involved in the pathogenesis of several diabetic complications. As a downstream target of the MAPK pathway, NF-κB activation was linked to pancreatic β-cell dysfunction [43,44]. In this study, DL-arginine treatment significantly reduced the pancreas’ *MAPK* and *NF-κB* expression and subsequent inflammatory response. Similarly, DL-arginine treatment reduced the *MAPK* expression and subsequent inflammatory response in the thymus, which is both an immune and endocrine organ [45]. Notably, in the AGEs production-restricted group, the delay or absence of IR is reportedly linked to low oxidative stress and high AGER1 levels [46]. Oxidative stress is known to be involved in the development of IR. DL-arginine can also inhibit the inflammatory response in the liver by reducing the expression of *AGER1*. Thus, DL-arginine potentially exerts protective effects by preventing the production of deleterious cytokines involved in the progression of diabetic complications through the inhibition of NF-κB, MAPK, and AGER1 signaling pathway activation.

Abnormalities in glucose metabolism and diabetes are one of the greatest health concerns in the world. To have inexpensive and effective interventions that are dietary and have been employed by many humans to establish a great safety profile, all recommend arginine as a potential agent for improving human health. It has been proved the administration of L-arginine could not only delay the development of T2DM for a long period but also improve peripheral and hepatic insulin sensitivity in T2DM patients [22,23]. Our finding also demonstrated that DL-arginine regulated hyperglycemia tolerance to relieve tissue injury, hinting that DL-arginine might be a novel target for developing therapeutic agents to regulate hyperglycemia.

## 4. Materials and Methods

### 4.1. Ethics Statement

All animal management and handling procedures were approved by the Yangzhou Institutional Animal Committee (Permit Number: 202106001, 1 June 2021, Yangzhou University, Government of Jiangsu Province, China).

### 4.2. Experimental Animals

A total of eighty 8-week-old Sprague–Dawley (SD) rats (male, body weights: 300–350 g) were from the Animal Center of Nantong University (Nantong, China). During this experiment, the healthy rats were kept in a 20-square-foot room (20 rat cages, 4 rats per cage) and were maintained under 12/12 h light/dark conditions at an ambient temperature of 22–25 °C. The rats were randomized into two groups, normal rats (*n* = 12) were fed a standard diet, while the other rats (*n* = 68) were fed a 45% high-fat diet (HFD) for 4 weeks (the diet composition is listed in Supplementary Tables S1 and S2).

A total of two hundred and eighty 7-day-old white broiler chickens (male, body weights: 350–380 g) were from the Suqian Hongwei Chicken Farm (Suqian, China). The healthy chickens were kept in an 18.5-square-foot room (47 wire cages, 4–6 chickens per cage) and were maintained under 16/8 h light/dark conditions at an ambient temperature of 24–27 °C for 7 days. During this experiment, the chickens were fed ad libitum with wheat, corn, and soya meal (Supplementary Table S3). After 7 days of acclimatization, sixty chickens were induced by insulin or glucose administration. In addition, two hundred and twenty chickens collect chicken serum for treating STZ-induced T2DM rats.

### 4.3. STZ-Induced T2DM Rats

The sixty-eight 12-week -old rats fasted for 12 h with free access to water before the experiment, and HFD-fed rats were intraperitoneally injected with STZ (35 mg/kg in 0.1 M citrate-buffered saline, pH 4.5) to induce T2DM (Sigma-Aldrich, St. Louis, MO, USA) [47]. STZ-treated rats were fed with HFD for another week and then subjected to 12 h of fasting before the blood glucose test. Blood glucose levels were determined using a glucometer (ACCU-CHEKA Performa; Roche Diagnostics GmbH, Mannheim, Germany). The rats that showed fasting glucose levels >16.7 mmol/L were considered T2DM rats. STZ-induced T2DM rats were anesthetized with sodium pentobarbital, and humanely euthanized, and pancreas samples were collected for hematoxylin/eosin-stained (HE) sections.

### 4.4. Acute Hypoglycemic and Hyperglycemic Chickens Induced by Insulin/Glucose

The sixty 14-day-old chickens fasted for 12 h with free access to water before the experiment and were stochastically allocated into three groups (*n* = 20 per group). The acute hypoglycemic chickens were subcutaneously injected with insulin (2 IU/kg BW), the acute hyperglycemic chickens were orally administered glucose (2 g/kg BW), and the control birds were subcutaneously injected with 2 mL of normal saline. The blood glucose levels were determined at 0, 30, 60, and 120 min using a glucometer (Roche Diagnostics GmbH). They successfully insulin/glucose-induced in acute hypoglycemic chickens for 60 min, acute hyperglycemic chickens for 30 min, and control birds for 60 min. Serum samples of acute hypoglycemic/hyperglycemic chickens and control birds were collected for untargeted metabolomics (*n* = 6 per group). After labeling, samples were quickly frozen in liquid nitrogen for 15 min and cryopreserved at −80 °C for future use. The chickens were anesthetized with sodium pentobarbital and humanely euthanized, and pancreas samples were collected for HE sections and immunohistochemistry.

### 4.5. HE Sections and Immunohistochemistry

The rat liver, kidney, and pancreas and chicken pancreas were fixed with 4% paraformaldehyde, gradually dehydrated, embedded in paraffin, cut into 4 μm-thick sections, and stained with HE (Servicebio Technology Co., Ltd., Wuhan, China). Immunohistochemical staining was performed according to the manufacturer’s instructions (Servicebio Technology Co., Ltd.). Chicken pancreas samples were fixed in 4% paraformaldehyde for 24 h and paraffin-embedded. Serial pancreas sections (10 mm in thickness) were sliced from each sample using a cryostat (CM 1860, Leica Biosystems, Wetzlar, Germany), and blocked with 10% normal goat serum (Santa Cruz Biotechnology, Inc., Santa Cruz, CA, USA). Sections were incubated overnight with anti-IRS1 primary antibody (Bioss Biotech Co., Ltd., Beijing, China) at 37 °C, followed by incubation with secondary goat anti-mouse IgG H&L (HRP) antibody (Servicebio Technology Co., Ltd.) for 30 min at 37 °C. Representative areas were photographed using a Nikon 90i microscope (Nikon, Tokyo, Japan).

### 4.6. Chicken Serum Treatment in STZ-Induced T2DM Rats

The two hundred and twenty 14-day-old chickens fasted for 12 h with free access to water before the experiment. The 3 mL blood was collected from each chicken into coagulation-promoting tubes and allowed to stand at 4 °C for 1 h for coagulation stratification (Huabo Medical Device Co., Ltd., Chengwu, China). Then, about 1 mL of chicken serum was separated from each chicken’s blood via centrifugation at 3000× *g* for 10 min at room temperature. A total of 220 mL serum was collected and stored at −80 °C for future use.

The STZ-induced T2DM rats were randomly allotted to 4 treatments (*n* = 6 per treatment): Normal + PBS group, STZ-induced + PBS group, STZ-induced + insulin group, and STZ-induced + serum group. The rats in the STZ-induced + insulin group received subcutaneous injections of insulin (2 IU/kg) daily. The rats in the STZ-induced + serum group were subcutaneous injection with chicken serum (1 mL/kg) daily. The treatment lasted 10 weeks, and the feed intake, body weight, and blood glucose of the rats were monitored.

### 4.7. Measurement of the Serum AGEs, Fructosamine and Biochemical Blood Examination

After the last subcutaneous injection of chicken serum, the serum fructosamine and AGE levels were also measured. Fructosamine and AGE levels were measured using an ELISA kit (Meimian Industrial Co., Ltd., Yancheng, China), following the manufacturer’s instructions. In addition, the serum aspartate aminotransferase, alanine aminotransferase, and alkaline phosphatase levels were measured by fully automated instruments for measuring biochemical indicators (Beckman Coulter, AU480, Brea, CA, USA).

### 4.8. Untargeted Metabolomics of Chicken Serum

#### 4.8.1. Metabolite Extraction

Chicken serum sample (100 μL) was collected into 1.5 mL EP tubes (Axygen Scientific, Union, CA, USA), following which 400 μL of 80% methanol aqueous solution was added and vortexed, placed in an ice bath for 5 min, and finally centrifuged at 15,000× *g* and 4 °C for 20 min. Then, a certain amount of supernatant was diluted with mass spectrometry grade water to 53% methanol content and centrifuged at 15,000× *g* and 4 °C for 20 min; the supernatant was collected and injected into the LC-MS/MS system for analysis [48,49].

#### 4.8.2. UHPLC-MS/MS Analysis

UHPLC-MS/MS analyses were performed using a Vanquish UHPLC system (Thermo Fisher Scientific, Waltham, MA, USA), coupled with an Orbitrap Q Exactive^TM^ HF mass spectrometer (Thermo Fisher Scientific, Waltham, MA, USA) in (Novogene Co., Ltd., Beijing, China). Samples were injected into a Hypersil Gold column (100 × 2.1 mm, 1.9 μm) at a flow rate of 0.2 mL/min and a linear gradient of 17 min. The positive mode eluent was A (0.1% FA aqueous solution) and B (methanol), while the negative mode eluent was eluent A (5 mM ammonium acetate, pH 9.0) and B (methanol). The solvent gradient was set as follows: 2% B, 1.5 min; 2–100% B, 12.0 min; 100% B, 14.0 min; 100–2% B, 14.1 min; the Q Exactive^TM^ HF mass spectrometer operates in a positive/negative polarity mode, with a spray voltage of 3.2 kV, a capillary temperature of 320 °C, a sheath gas flow rate of 40 arb, and an auxiliary gas flow rate of 10 arb.

#### 4.8.3. Data Processing and Metabolite Identification

The lower machine data (.raw) files were imported into the CD3.1 (CD3.1, Thermo Fisher) search software for processing; each metabolite was screened for parameters such as retention time and mass-to-charge ratio. A retention time deviation of 0.2 min and mass deviation of 5 ppm was set for peak alignment of different samples for accurate identification. Peak extraction was conducted with information such as mass deviation of 5 ppm, signal intensity deviation of 30%, signal-to-noise ratio of 3, and minimum signal intensity. The peak area was quantified, and the target ions were integrated, followed by a molecular formula prediction through molecular ion peaks and fragment ions with mzCloud (https://www.mzcloud.org, accessed on 14 September 2021). The mzVault and MassList databases were compared, background ions were removed using blank samples, the original quantitative results were normalized, and metabolite identification and relative quantitative results were generated. Data processing was conducted using the Linux operating system (CentOS version 6.6) as well as software R and Python; the specific package and software version are indicated in the result file readme.

#### 4.8.4. Data Analysis

Using the KEGG [50,51] (https://www.genome.jp/kegg/pathway.html, accessed on 14 September 2021), HMDB (https://hmdb.ca/metabolites, accessed on 14 September 2021), and the LIPID Maps databases (http://www.lipidmaps.org, accessed on 14 September 2021), the identified metabolites were annotated. In the multivariate statistical analysis potion, principal component analysis (PCA) and partial least squares discriminant analysis (PLS-DA) were performed after data transformation using the metabolomics data processing software metaX. The VIP values were obtained for each metabolite. Additionally, in the univariate analysis portion, the statistical significance (*p*-value) of each metabolite between the two groups was calculated using the *t*-test; the fold change (FC value) of metabolites between the two groups was also calculated. The default criteria for the differential metabolite screening were VIP > 1, *p* < 0.05, and FC ≥ 2 or FC ≤ 0.5. The volcano plot was generated using the R package ggplot2 [52], which can generate the values of three parameters, namely, VIP value, log2 (Fold Change), and −log10 (*p*-value) of metabolites to screen the metabolites of interest. The cluster heatmap was plotted using the R package Pheatmap [53], while the metabolite data were normalized using the z-score. Correlation analysis (Pearson correlation coefficient) between the differential metabolites was performed using the cor () function of the R language [54] with statistical significance via R language cor.Mtest (); *p* < 0.05 was considered statistically significant, and correlations were plotted using the corrplot package in R language [55]. Bubble plots were generated using the R package ggplot2; the KEGG database was used to investigate the function and metabolic pathways of the metabolites, which were considered enriched when x/*n* > y/*n*, and significantly enriched when the *p*-value of the metabolic pathway was < 0.05.

### 4.9. DL-Arginine Treatment in STZ-Induced T2DM Rats

**Scheme 1:**
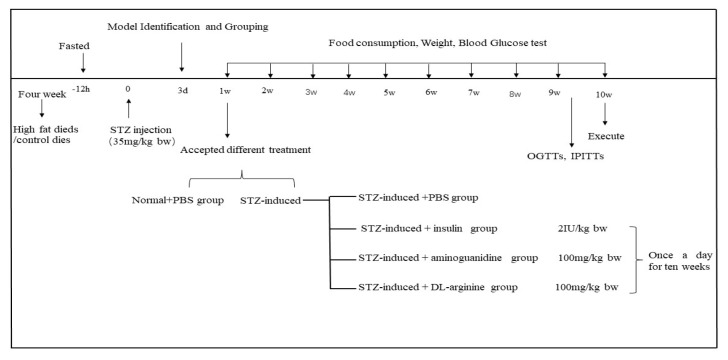
Time point of different treatments of animal models.

The STZ-induced T2DM rats were randomly allotted to five treatments (*n* = 6 per treatment): Normal + PBS group, STZ-induced + PBS group, STZ-induced + insulin group, STZ-induced + aminoguanidine group, and STZ-induced + DL-arginine (Sigma-Aldrich, St. Louis, MO, USA) group. The rats in the STZ-induced + insulin group received subcutaneous injections of insulin (2 IU/kg). Aminoguanidine and DL-arginine were dissolved in 1 mL phosphate-buffered saline and orally administered to STZ-induced T2DM rats (100 mg/kg BW). The treatment was performed once daily for 10 weeks (see Scheme 1 for the detailed experimental procedures.) The body weight and feed intake of rats in each group were recorded and calculated every week, and the rats were fasted for 3 h before measuring the blood glucose levels. Tail capillary blood glucose levels were monitored using a glucometer (Roche Diagnostics GmbH). Fructosamine and AGE levels were measured as in the previous chicken serum treatment. After 10 weeks of treatment, STZ-induced T2DM rats were anesthetized with sodium pentobarbital and humanely euthanized. Skeletal muscle, liver, pancreas, and thymus glands were collected quickly in a freezing tube, frozen in liquid nitrogen, and stored at −80 °C for real-time qRT-PCR. In addition, the liver, kidney, and pancreas were also collected for HE sections. The HE section protocol was the same as previously mentioned.

### 4.10. OGTTs, IPITTs

For OGTTs, rats were fasted overnight, following which glucose (2 g/kg BW) was orally administered. Blood glucose levels were measured at 0, 30, 60, 90, and 120 min post-dosing using a glucometer (Roche Diagnostics GmbH). For IPITTs, rats were fasted overnight and orally administered glucose (2 g/kg BW) immediately, followed by the subcutaneous injection of insulin (2 IU/kg BW) for IPITTs. Blood glucose levels were subsequently measured at 0, 30, 60, 90, and 120 min post-dosing using a glucometer (Roche Diagnostics GmbH). The IR index was calculated using the following formula: HOMA-IR index = (FBG [mmol/L] × FINS [units/L])/22.5 [56].

### 4.11. Real-Time qRT-PCR

Total RNA was extracted from the skeletal muscle, liver, pancreas, and thymus glands. Primers were designed using the Primer 5.0 software and synthesized (Tsingke Biotechnology Co., Ltd., Nanjing, China) (Supplementary Table S4). The reaction was performed using ChamQ Universal SYBR qPCR Master Mix (Vazyme Biotechnology Co., Ltd., Nanjing, China). Three samples were selected for each tissue in each group, with three replicates for each sample. The β-actin gene was used as the reference gene to normalize the expression data, which was calculated with the 2^−ΔΔCT^ method [57].

### 4.12. Statistical Analysis

Pairwise comparisons between different groups were conducted using SPSS statistical software (version 18.0; SPSS, Chicago, IL, USA). Data are presented as means  ±  standard error (SE). The peak representative areas were compared using a Student’s *t*-test. Blood glucose levels were compared using a Student’s *t*-test or one-way ANOVA. The body weight, feed intake, insulin content, area under the curve, HOMA-IR coefficient, fructosamine and AGEs levels, and quantitative data were compared using one-way ANOVA. OGTTs and IPITTs were compared using repeat-measure ANOVA. Mapping was performed using Prism software (GraphPad, San Diego, CA, USA). Statistical significance was set at *p* < 0.05.

## 5. Conclusions

In summary, we report that DL-arginine in chicken serum was identified as a potential marker for the homeostatic regulation of hyperglycemia in chickens. DL-arginine could reduce both blood glucose and AGEs levels, thereby promoting the expression of inflammatory factors and relieving tissue injury in STZ-induced T2DM rats. Therefore, our study provides a basis for applying DL-arginine as a potential therapeutic target for T2DM.

## Figures and Tables

**Table 1:** Screening results of differential metabolites.

Compared Samples	Num. of Total Ident.	Num. of Total Sig.	Num. of Sig. Up	Num. of Sig. Down
Glucose. vs. Control_ pos	429	124	84	40
Glucose. vs. Control_ neg	295	125	96	29
insulin. vs. Control_ pos	429	118	73	45
insulin. vs. Control_ neg	295	79	33	46

## Data Availability

The data presented in this study are available on request from the corresponding author.

## Supplementary Materials

The following supporting information can be downloaded at: https://www.mdpi.com/article/10.3390/ijms24010523/s1.
