# Supplementary material for: Effects of Chicken Serum Metabolite Treatment on the Blood Glucose Control and Inflammatory Response in Streptozotocin-Induced Type 2 Diabetes Mellitus Rats
Source: Int J Mol Sci. 2022 Dec 28;24(1):523. doi: 10.3390/ijms24010523 (PMC9820086; doi:10.3390/ijms24010523)
Supplement: Supplementary file 1 [file ijms-24-00523-s001.zip › ijms-1995731-supplementary.pdf]

Supporting Information

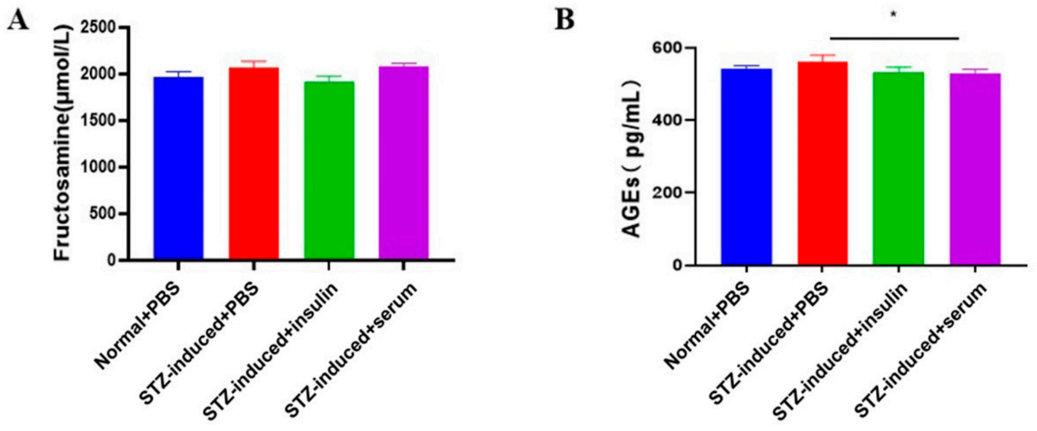

Figure S1 Effect of chicken serum on fructosamine and AGE levels in rat serum. (**A**) Individual fructosamine levels in four groups (Normal + PBS, STZ-induced + PBS, STZ-induced + insulin, STZ-induced + serum) were evaluated by ELISA. (**B**) Individual AGEs levels in four groups (Normal + PBS, STZ-induced + PBS, STZ-induced + insulin, STZ-induced + serum) were evaluated by ELISA. All values of a, b are mean ± SE *n*=6 rats per group, with *$P < 0.05$ determined by one-way ANOVA.

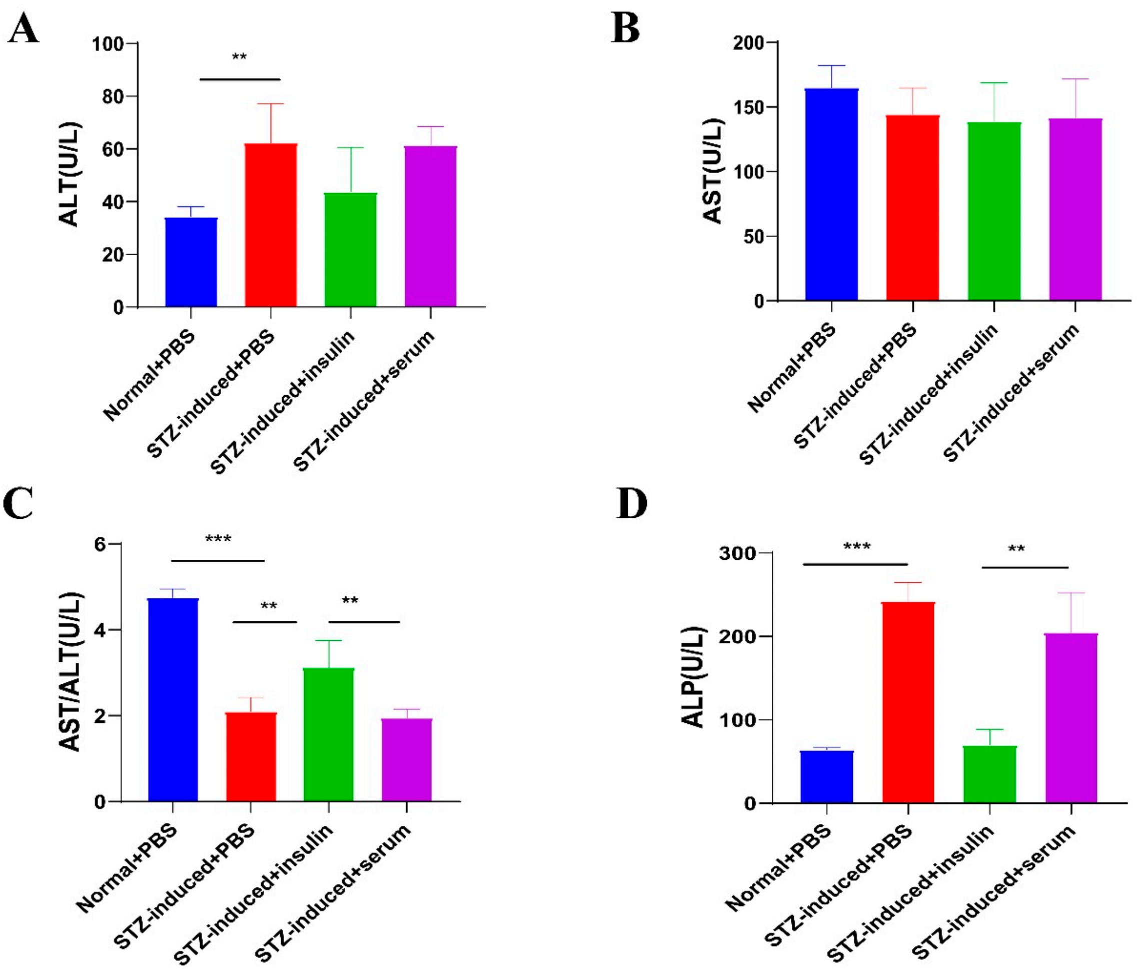

Figure S2. Effect of chicken serum on biochemical detection in rat serum. (**A**) Individual alanine transferase levels in four groups (Normal + PBS, STZ-induced + PBS, STZ-induced + insulin, STZ-induced + serum) were evaluated by serum biochemical examination. (**B**) Individual aspartate aminotransferase levels in four groups (Normal + PBS, STZ-induced + PBS, STZ-induced + insulin, STZ-induced + serum) were evaluated by serum biochemical examination. (**C**) Individual aspartate/aspartate alanine levels in four groups (Normal + PBS, STZ-induced + PBS, STZ-induced + insulin, STZ-induced + serum) were evaluated by serum biochemical examination. (**D**) Individual alkaline phosphatase levels in four groups (Normal + PBS, STZ-induced + PBS, STZ-induced + insulin, STZ-induced + serum) were evaluated by serum biochemical examination. All values of a-d are mean ± SE n=6 rats per group, with $^{**}P < 0.01$,$^{***}P < 0.001$ determined by one-way ANOVA.

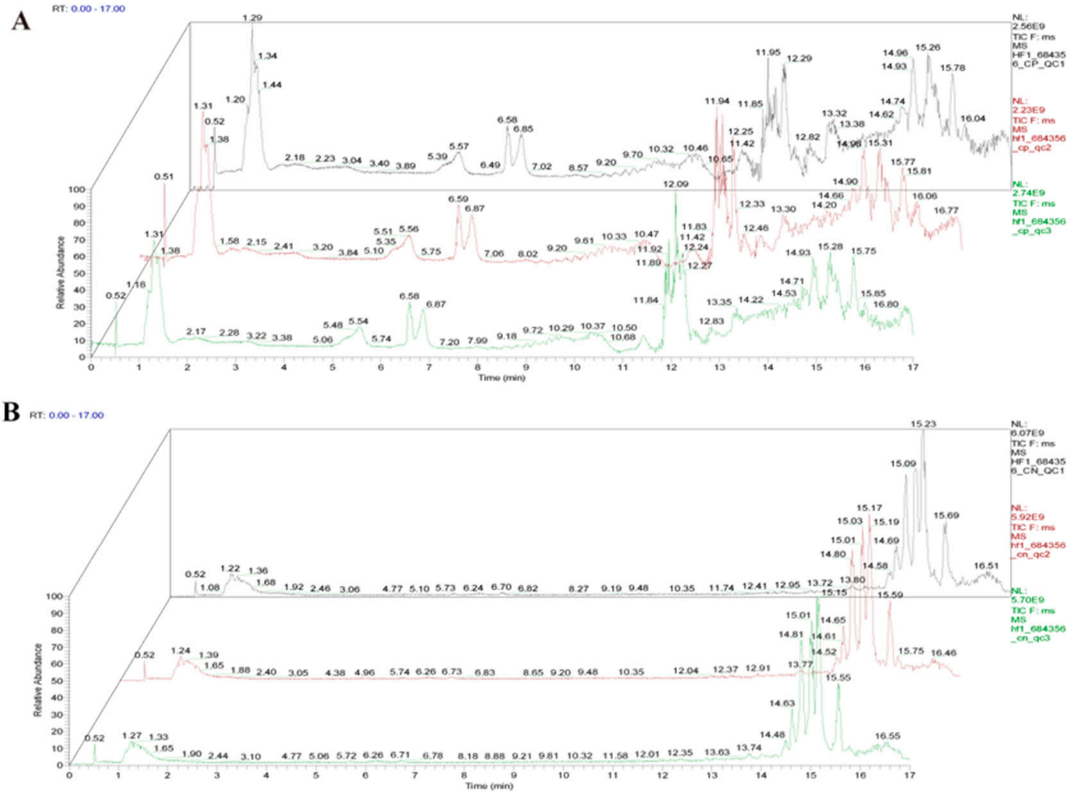

Figure S3. Total ion current (TIC) chromatograms of metabolites in acute hyperglycemic/hypoglycemic chickens and control birds. (**A**) positive ion mode. (**B**) negative ion mode.

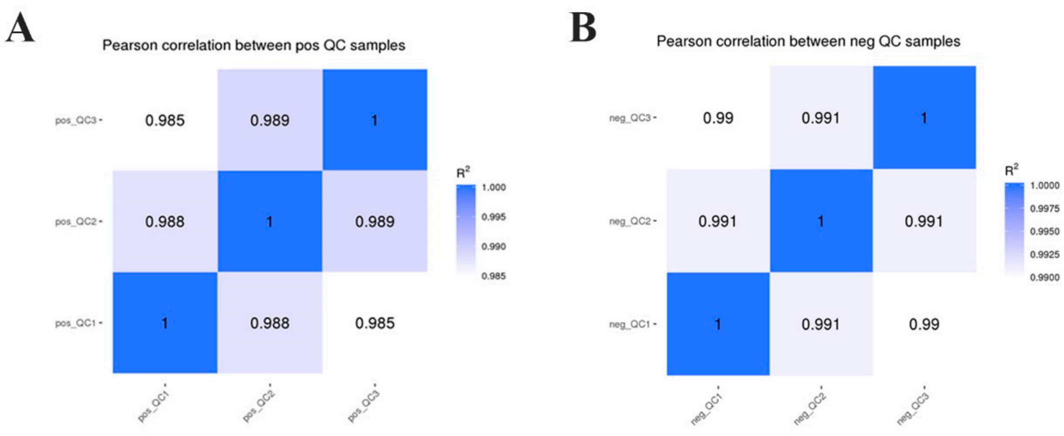

Figure S4. Correlation analysis of QC samples of serum metabolites in acute hyperglycemic/hypoglycemic chickens and control birds. (**A**) positive ion mode. (**B**) negative ion mode.

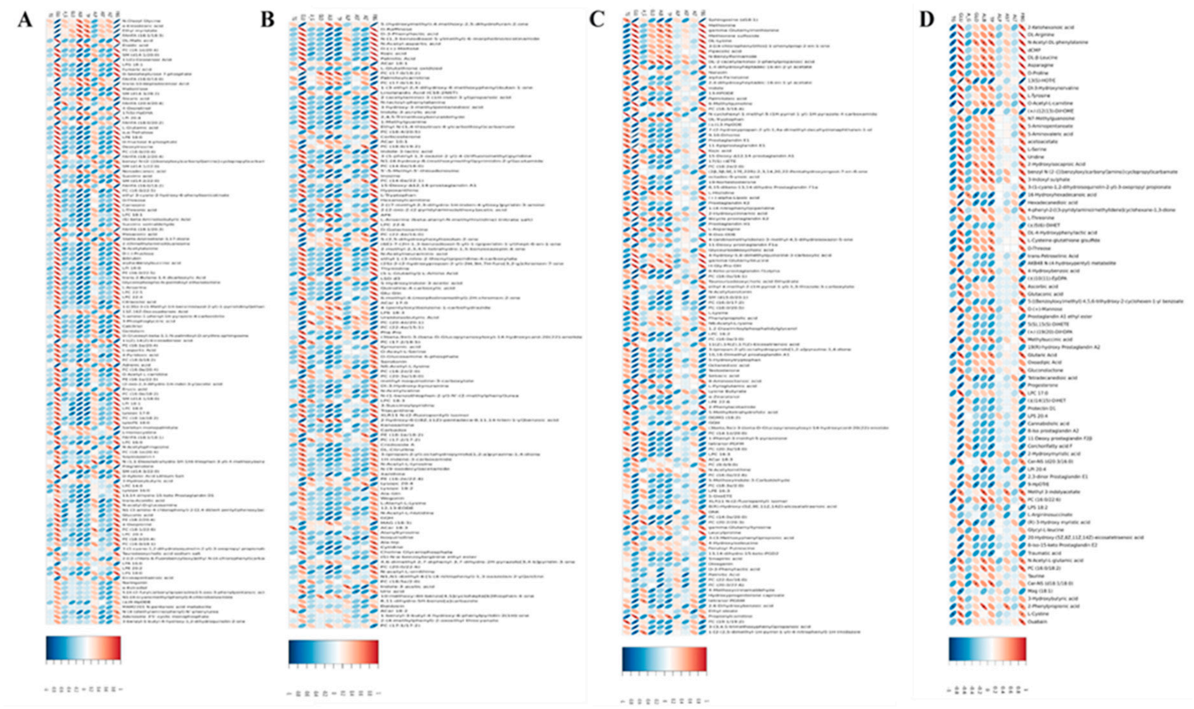

Figure S5. Correlation analysis of differential metabolites between acute hyperglycemic/hypoglycemic chickens and control birds. (**A**) Acute hyperglycemic chickens and control birds positive ion mode. (**B**) Acute hyperglycemic chickens and control birds negative ion mode. (**C**) Acute hypoglycemic chickens and control birds positive ion mode. (**D**) Acute hypoglycemic chickens and control birds negative ion mode.

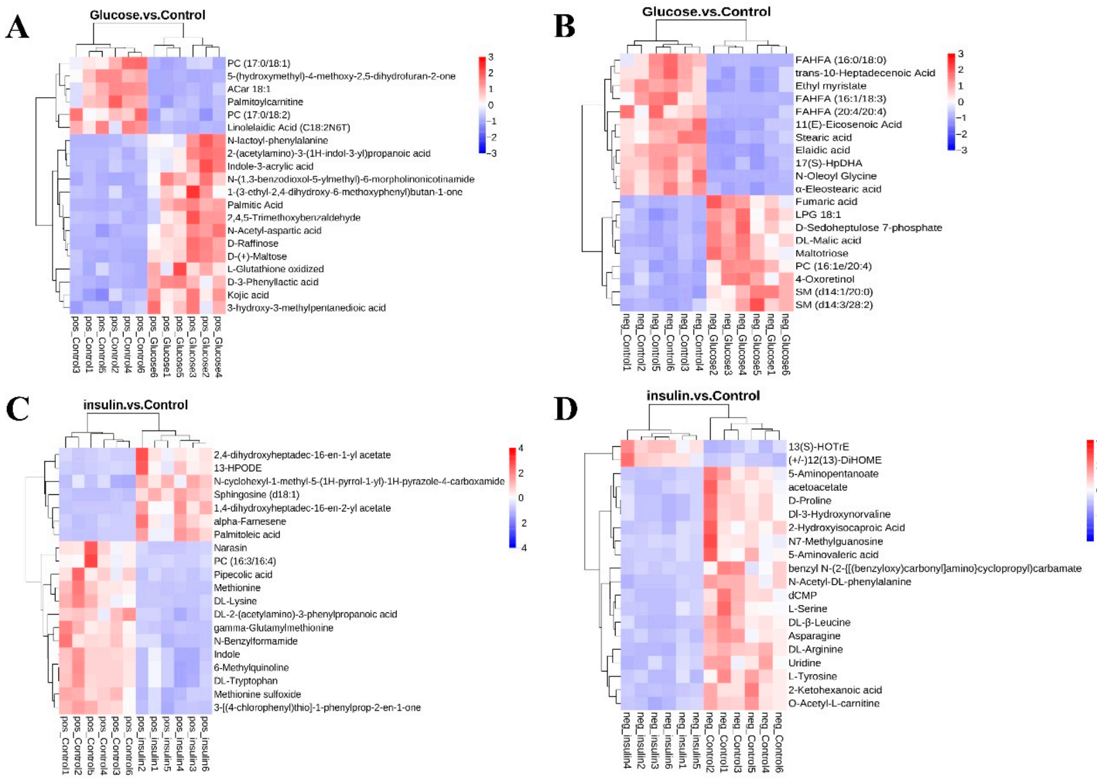

Figure S6. Heat map of differential metabolite clusters in acute hyperglycemic/hypoglycemic chickens and control birds. (**A**) Acute hyperglycemic chickens and control birds positive ion mode. (**B**) Acute hyperglycemic chickens and control birds negative ion mode. (**C**) Acute hypoglycemic chickens and control birds positive ion mode. (**D**) Acute hypoglycemic chickens and control birds negative ion mode. The horizontal rows in the figure represent the samples of each group, the vertical columns represent the differential metabolites, the red represents the up-regulated expression of the differential metabolites, and the blue represents the down-regulated expression of the differential metabolites. The darker the color, the more obvious the difference.

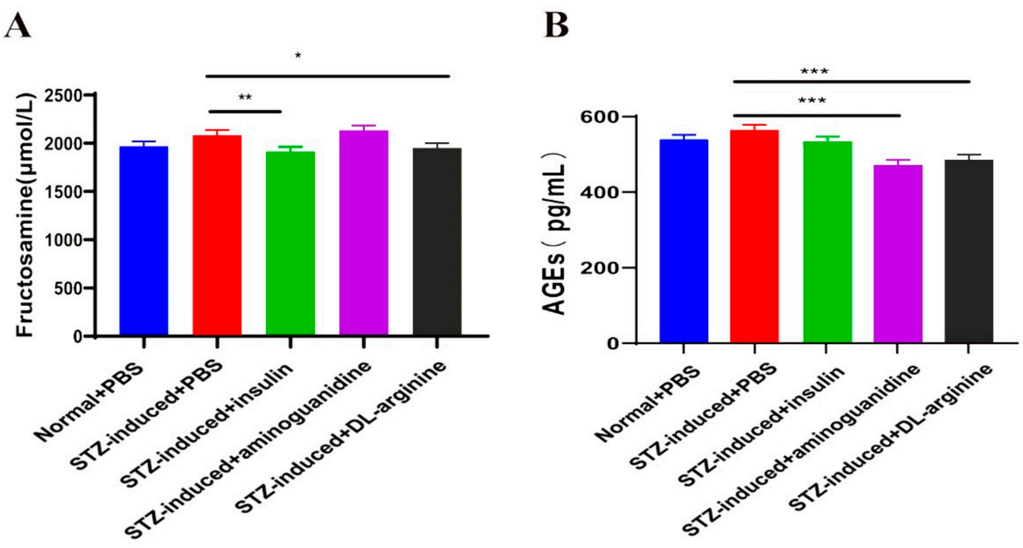

Figure S7. Effect of DL- arginine on fructosamine and AGEs levels in rat serum. (**A**) Individual fructosamine levels in five groups (Normal + PBS, STZ-induced + PBS, STZ-induced + insulin, STZ-induced + aminoguanidine, STZ-induced +DL-arginine) were evaluated by ELISA. (**B**) Individual AGEs levels in five groups (Normal + PBS, STZ-induced + PBS, STZ-induced + insulin, STZ-induced + aminoguanidine, STZ-induced +DL-arginine) were evaluated by ELISA. All values of a,b are mean ± SE n=6 rats per group, with *$P < 0.05$, **$P < 0.01$,***$P < 0.001$ determined by one-way ANOVA.

Table S1. Rat standard diet composition.

Ingredient	gm%	Protein, %	Subtotal	Fat, %	Subtotal	Fiber, %	Subtotal	Ash, %	Subtotal	Calcium, %	Subtotal	Phosphorus, %	Subtotal
Basic diet	1	18.27	18.27	4.8	4.8	3.57	3.57	5.89	5.89	1.22	1.22	0.839	0.839
Total	1		18.27		4.8		3.57		5.89		1.22		0.839
										Energetic substance	gm, %	Kcal/kg	Kcal, %
										Protein	18.27	730.8	21.11%
										Fat	4.8	432	12.48%
										Carbohydrate	57.47	2298.8	66.41%
											Total energy	3461.6	100.00%

Table S2. Rat 45% high-fat diet (HFD) composition.

Ingredient	gm%	Protein,%	Subtotal	Fat,%	Subtotal	Fiber,%	Subtotal	Ash,%	Subtotal	Calcium,%	Subtotal	Phosphorus,%	Subtotal
Basic diet	0.435	19	8.265	5	2.175	3	1.305	5.5	2.3925	1	0.435	0.78	0.3393
Lard	0.175	0	0	98	17.15	0	0	0	0	0	0	0	0
Sucrose	0.12	0	0	0	0	0	0	0.5	0.06	0.04	0.0048	0.01	0.0012
Whole milk powder	0.1	40	4	48	4.8	0	0	0	0	0	0	0	0
Casein	0.13	90	11.7	0	0	0	0	0	0	0	0	0	0
Mineral/Vitamin Mix	0.02	22	0.44	1.4	0.028	0.8	0.016	25	0.5	7.18	0.1436	5.25	0.105
Calcium hydrogen phosphate	0.02	0	0	0	0	0	0	78.48	1.5696	22	0.44	17	0.34
Total	1		24.405		24.153		1.321		4.5221		1.0234		0.7855
										Energetic substance	gm,%	Kcal/kg	Kcal,%
										Protein	24.41	976.2	21.34%
										Fat	24.15	2173.77	47.53%
										Carbohydrate	35.6	1423.96	31.13%
											Total energy	4573.926	100.00%

TableS3. Chicken diet composition.

Ingredient (%)		Calculated Nutrient	
Corn (%)	58.04	Granulometry (μ)	1,362.54
Soya (%)	10	Crude protein (%)	21
Soybean cake granule (%)	13.1	AMEn poultry (kcal/kg)	2,989.03
Dresses distillery (%)	5	Phosphorus available (%)	0.5
Corn gluten (%)	4.6	Total chloride (%)	0.21
Canola oil cake (%)	4.8	Total sodium (%)	0.22
Limestone (%)	1.53	Choline added (mg/kg)	396.99
Monocalcium phosphate (%)	1.25	Vit.A added (IU/kg)	11,000.00
Soybean oil (%)	-	Vit.D added (IU/kg)	4,988.24
Lysine sulfate 70% (%)	0.43	Vit.E added (IU/kg)	80
Sodium bicarbonate (%)	0.36	Arg Dig V Vol (%)	1.16
Salt (%)	0.25	SID Lys (%)	1.08
Luzern concentrate (%)	0.2	SID Met and Cys (%)	0.8
Methionine (%)	0.18	SID Met (%)	0.49
Myco-curb liquid (%)	0.1	SID Thr (%)	0.67
Choline liquid 75% (%)	0.06	SID Try (%)	0.21
Hy D premix (Vitamin D3) (%)	0.03	Calcium (%)	1
Threonine 98% (%)	-		
OptiPhos 1000ct 250 ftu (0.12%) (%)	0.03		
Vitamin E 100,000 IU (%)	0.05		

Table S4. Sequences of real-time PCR primers.

Primer name	primer sequence5'→3'	Annealing temperature °C
β-actin-F	GACCTGTACGCCAACACAGT	60°C
β-actin-R	CTCAGGAGGAGCAATGATCT	
CS-F	GGAGCCAAGAACTCATC	60°C
CS-R	CCACCGTACATCATGTC	
GCK-F	CCACAATCATGCCGACCTCA	60°C
GCK-R	ATGCAGCACCTGATAGC	
PKM-F	ATGCAGCACCTGATAGC	60°C
PKM-R	TGCGTGTCACAGCAATG	
ICDH-F	GTGTATAACTTCCCTGCCGGA	60°C
ICDH-R	AGCGGCCATTTCTTCTGGAT	
GLUT2 F	CACCAGCACATACGACACCAGAC	60°C
GLUT2 R	CCCAAGCCACCCACCAAAGAAC	
GLUT4-F	GTGCCTATGTATGTGGGAGAA	60°C
GLUT4-R	TGCAGGAGAGCAGGGAGTA	
RAGE-F	CACGAGGATGAGGGCACCTA	60°C
RAGE-R	CCTCATCGCCGGTTTCTGTGA	
AGER1-F	CTGCCTCTGAACTCACAGCCAATG	60°C
AGER1-R	TCCTGGTCTCCTCCTTCACAACTG	
MAPK-F	CTACTGGGCTCCAAGCCAAA	60°C
MAPK-R	CTAAAACTCTGCAGCGCGTC	
NF-κB-F	TGTGGTGGAGGACTTGCTGAGG	60°C
NF-κB-R	AGTGCTGCCTTGCTGTTCTTGAG	
